# Supplementary material for: Prognostic value of high-sensitivity cardiac troponin for major adverse cardiovascular events in patients with diabetes: a systematic review and meta-analysis
Source: PeerJ. 2023 Nov 13;11:e16376. doi: 10.7717/peerj.16376 (PMC10652853; doi:10.7717/peerj.16376)
Supplement: Supplemental Information 1 — hs-cTn =high-sensitivity cardiac troponin, HR =hazard ratio, SD =standard deviation, OR =odds ratio, IQR =interquartile range, MACE =major adverse cardiovascular events, IHD =ischemic heart diseases, CVD =cardiovascular diseases, CAD =coronary artery disease, NA =not available. a, data was presented as mean ±standard deviation or median (interquartile range). [file peerj-11-16376-s001.doc]

**Supplementary Table 1. Supplementary information of included studies.**

| Author | Year | Age (years) | Male (%) | Coding of hs-cTn | Patient source | Outcomes of interest | Adjusted covariates |
| --- | --- | --- | --- | --- | --- | --- | --- |
| Witkowski | 2021 | 65±11.2 | 72.4 | Quartiles (8.2/13/21.6 ng/L) | Cleveland Clinic GeneBank Study (2001-2007) | MACE, all-cause mortality | Demographics, cardiovascular risk factors |
| Saeed | 2021 | 32.7 (20.9–44.0) | 54 | Continuous (HR per SD) | Nationwide Norway study (2002-2003) | Incidence IHD event | Systolic blood pressure, albuminuria, and HbA1c |
| Pandey | 2021 | 57 (51-63) | 51.2 | Continuous (HR per SD); binary (6 ng/L) | ARIC study (1990-1992), DHS study (2000-2002), MESA study (2000-2002) | Heart failure | Demographics, cardiovascular risk factors, medication use, study cohort, biomarker levels |
| Pandey | 2021 | 58 (53-64) | 46.8 | Continuous (HR per SD); binary (6 ng/L) | ARIC study (1990-1992), DHS study (2000-2002), MESA study (2000-2002) | Heart failure | Demographics, cardiovascular risk factors, medication use, study cohort, biomarker levels |
| Cimaglia | 2021 | 73 (65-80) | 72 | Binary (≥25 ng/L） | Maria Cecilia Hospital (Cotignola, Italy) | MACE, all-cause mortality | Comorbidities |
| Busch | 2021 | 65.5 (58.8-71.4) | 65.9 | Continuous (HR per SD) | Thousand&2 study | MACE | NA |
| Bluro | 2021 | 65 (55-72) | 56.3 | Continuous (HR per SD); Binary (14ng/L) | Hospital Italiano de Buenos Aires (2013-2017) | MACE, all-cause mortality, stroke | Age, eGFR |
| Tang | 2020 | 75.5±5.1 | 43 | Continuous (HR per log2); binary (≥9.2 ng/L） | ARIC study (2011-2013) | All-cause mortality | NA |
| Tang | 2020 | 75.5±5.1 | 43 | Continuous (HR per log2); binary (≥24 ng/L） | ARIC study (2011-2013) | All-cause mortality | NA |
| Sharma | 2020 | 60.9±9.9 | 67.7 | Continuous (HR per log2) | EXAMINE trial | MACE | Demographics, cardiovascular risk factors, biomarker levels |
| Nguyen | 2020 | 64.3±9.9 | 57.7 | Continuous (HR per SD); Binary (≥4.5 ng/L） | MESA study | Incidence IHD event, Heart failure | Demographics, cardiovascular risk factors, biomarker levels |
| Nguyen | 2020 | 64.8±9.6 | 52.5 | Continuous (HR per SD); Binary (≥4.5 ng/L） | MESA study | Incidence IHD event, Heart failure | Demographics, cardiovascular risk factors, biomarker levels |
| Costacou (gender subgroup) | 2020 | 28.4 (22.6, 34.8) | 48.1 | Continuous (HR per SD) | EDC study | MACE | Cardiovascular risk factors, biomarker levels |
| Wong | 2019 | 67.7 ± 10.5 | 72 | Continuous (HR per SD) | Cardiac Clinic, Queen Mary Hospital (2003-2014) | MACE | Demographics, cardiovascular risk factors, enrollment period |
| Keller | 2018 | 67±8 | 53.2 | Quartiles (35/55/90 ng/L) | German Diabetes and Dialysis Study (4D) | Incident CVD, all-cause mortality | cardiovascular risk factors, dialysis-related factors |
| Colombo | 2018 | 62.9 (39.2-77.3) | 67.6 | Continuous (HR per SD) | CARDS trial | Incident CVD | Demographics, cardiovascular risk factors, medication use, biomarker levels |
| Price | 2017 | 67.9±2.4 | 49.1 | Continuous (OR per SD) | ET2DS study | Incident CVD | Cardiovascular risk factors, biomarker levels |
| Ohkuma | 2017 | 67±7 | 60 | Continuous (HR per SD) | ADVANCE trial | Heart failure | Demographics, cardiovascular risk factors, medication use, biomarker levels |
| Galsgaard | 2017 | 45.4 (11.4) | 53 | Continuous (HR per doubling) | Steno Diabetes Center in Denmark (1993-2000) | Incident CVD, all-cause mortality | Demographics, cardiovascular risk factors, biomarker levels |
| Galsgaard | 2017 | 42.1 (10.5) | 61 | Continuous (HR per doubling) | Steno Diabetes Center in Denmark (1993-2000) | Incident CVD, all-cause mortality | Demographics, cardiovascular risk factors, biomarker levels |
| Bidadkosh | 2017 | 64±9 | 76 | Continuous (HR per doubling) | Sun-MACRO trial | Incidence CVD event | Demographics, cardiovascular risk factors, biomarker levels |
| Scirica | 2016 | 65.0± 8.5 | 66.9 | Continuous (HR per SD) | SAVOR-TIMI 53 study (2010-2013) | MACCE, Cardiovascular death,myocardial infarction, ischemic stroke, and hospitalization for heart failure (HF) | Demographics, cardiovascular risk factors, biomarker levels |
| Resl | 2016 | 55±13/66±10 | 56.3 | Continuous (HR per SD) | Vienna’s General Hospital and Hietzing Hospital Vienna (2005-2008) | Composite endpoint of unplanned hospitalisation for cardiovascular disease or death | Demographics, cardiovascular risk factors, biomarker levels |
| Junttila | 2016 | NA | NA | Binary (≥14 ng/L） | BARI study | Composite endpoint of cardiovascular death, myocardial infarction and stroke | A multitude of clinical variables including quantitative measure of CAD severity |
| Hendriks | 2016 | 67±12 | 45 | Continuous (HR per SD); tertiles(3/14 ng/L) | ZODIAC study（1998-2001） | All-cause mortality, cardiovascular mortality | Demographics, cardiovascular risk factors, medication use, biomarker levels |
| Gori | 2016 | 63±6 | 48 | Continuous (HR per SD) | ARIC study (1996–1998) | Incident CVD | Demographics, cardiovascular risk factors, medication use, biomarker levels |
| Looker | 2015 | 68.4 (61.3, 74.4)/68.8 (61.3, 76.5) | 57.1 | Continuous (OR per SD) | Go-DARTS study, SDR study, MONICA/KORA study, [IMT] IMPROVE study and Stockholm 60-year-old Study | Incident CVD | Demographics, cardiovascular risk factors, medication use, biomarker levels |
| Zellweger | 2015 | 70 (59–77) | 71.5 | Binary (14ng/L); Continuous (HR per SD) from KM curves | APACE study (2006-2012) | All-cause mortality | AMI, unstable angina, cardiac disease other than CAD, non-cardiac disease, and unknown cause of acute chest pain. |
| Hillis | 2014 | 66.89±6.61 | 61 | Continuous (HR per SD) | Preterax and Diamicron Modified Release Controlled Evaluation (ADVANCE) study | Cardiovascular events,all-cause mortality | Demographics, cardiovascular risk factors, medication use, biomarker levels |
| Yiu | 2014 | 64.4 ± 10.0 | 57 | Binary (male: 8.5 ng/L, female: 7.6 ng/L); Continuous (HR per SD) from KM curves | CDATS study | MACE, heart failure, myocardial infarction, cardiovascular mortality | Demographics, cardiovascular risk factors |

hs-cTn=high-sensitivity cardiac troponin, HR=hazard ratio, SD=standard deviation, OR=odds ratio, IQR=interquartile range, MACE=major adverse cardiovascular events, IHD=ischemic heart diseases, CVD=cardiovascular diseases, CAD=coronary artery disease, NA=not available.

^a^, data was presented as mean ± standard deviation or median (interquartile range).
