# Supplementary figures and images for: Prognostic value of high-sensitivity cardiac troponin for major adverse cardiovascular events in patients with diabetes: a systematic review and meta-analysis
Source: PeerJ. 2023 Nov 13;11:e16376. doi: 10.7717/peerj.16376 (PMC10652853; doi:10.7717/peerj.16376)

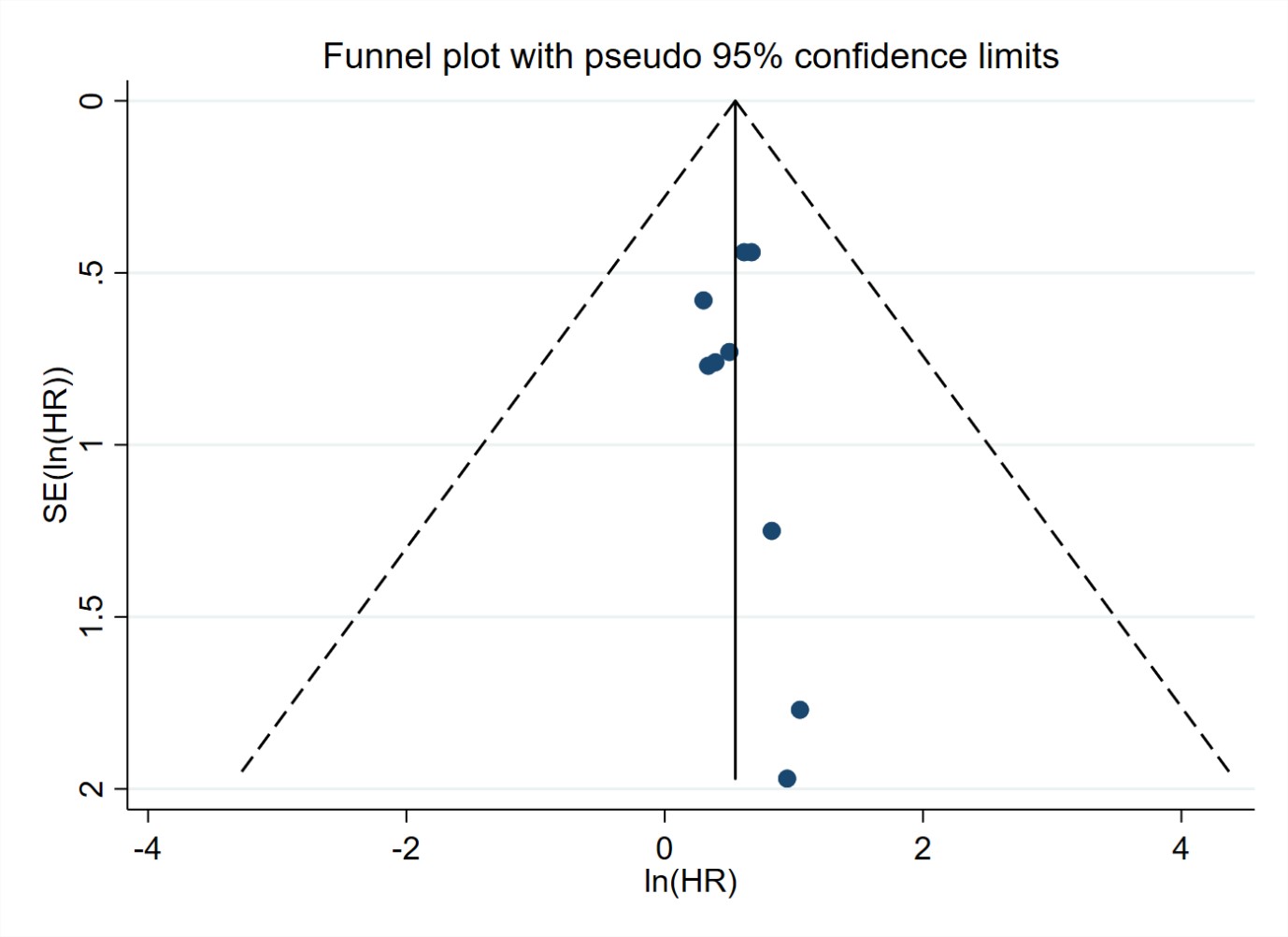

Supplement: Supplemental Information 2 [file peerj-11-16376-s002.jpg]

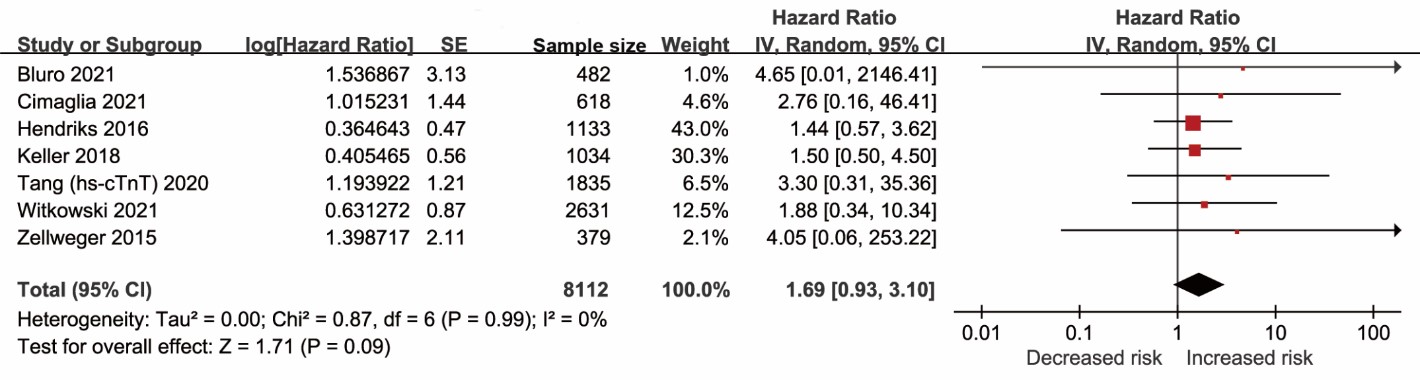

Supplement: Supplemental Information 3 [file peerj-11-16376-s003.jpg]
