# Supplementary material for: Prognostic value of high-sensitivity cardiac troponin for major adverse cardiovascular events in patients with diabetes: a systematic review and meta-analysis
Source: PeerJ. 2023 Nov 13;11:e16376. doi: 10.7717/peerj.16376 (PMC10652853; doi:10.7717/peerj.16376)
Supplement: Supplemental Information 7 [file peerj-11-16376-s007.docx]

**The rationale for conducting this systematic review and meta-analysis**

High-sensitivity cardiac troponin (hs-cTn) is associated with cardiovascular outcomes in general population, but the prognostic value of hs-cTn in diabetic population remains inconclusive. Presence of diabetes can innately raise the levels of cardiac biomarkers including hs-cTn, thus predictive value of hs-cTn in cardiovascular outcomes may be sheltered to some degree. To date, there are increasing prospective data on the use of hs-cTn in risk stratification in diabetic patients, but the results are inconclusive. To quantitatively summarize current evidence, we conducted a comprehensive systematic review and meta-analysis to evaluate the association between hs-cTn levels and risk of MACE as well as other adverse events in patients with diabetes or prediabetes.

**The contribution of this study to current evidence**

The prognostic value of high-sensitivity cardiac troponin (hs-cTn) has been established in general population, but its role in diabetes patients remains controversial. This systematic review and meta-analysis suggests hs-cTn also represents a strong and independent predictor of MACE and heart failure in patients diagnosed with diabetes. Our findings provided conceptual support to the expanded use of hs-cTn for predicting MACE and heart failure in population with diabetes. Future research is needed to determine the appropriate cutoff value for hs-cTn in diabetic population with different complications.
