# Supplementary material for: Prognostic value of high-sensitivity cardiac troponin for major adverse cardiovascular events in patients with diabetes: a systematic review and meta-analysis
Source: PeerJ. 2023 Nov 13;11:e16376. doi: 10.7717/peerj.16376 (PMC10652853; doi:10.7717/peerj.16376)
Supplement: Supplemental Information 9 — Literature search details [file peerj-11-16376-s009.doc]

**Appendix A. Meta-analysis Of Observational Studies in Epidemiology (MOOSE) checklist**

| **Item No** | **Recommendation** | **Reported on Page No** |
| --- | --- | --- |
| Reporting of background should include | | |
| 1 | Problem definition | 6 |
| 2 | Hypothesis statement | N/A |
| 3 | Description of study outcome(s) | 6 |
| 4 | Type of exposure or intervention used | 6 |
| 5 | Type of study designs used | 6 |
| 6 | Study population | 6 |
| Reporting of search strategy should include | | |
| 7 | Qualifications of searchers (eg, librarians and investigators) | 6 |
| 8 | Search strategy, including time period included in the synthesis and key words | 6 |
| 9 | Effort to include all available studies, including contact with authors | 6 |
| 10 | Databases and registries searched | 6-7 |
| 11 | Search software used, name and version, including special features used (eg, explosion) | Appendix 2 |
| 12 | Use of hand searching (eg, reference lists of obtained articles) | 7 |
| 13 | List of citations located and those excluded, including justification | Figure 1 |
| 14 | Method of addressing articles published in languages other than English | 6 |
| 15 | Method of handling abstracts and unpublished studies | Figure 1 |
| 16 | Description of any contact with authors | N/A |
| Reporting of methods should include | | |
| 17 | Description of relevance or appropriateness of studies assembled for assessing the hypothesis to be tested | 7-8 |
| 18 | Rationale for the selection and coding of data (eg, sound clinical principles or convenience) | 7 |
| 19 | Documentation of how data were classified and coded (eg, multiple raters, blinding and interrater reliability) | 7 |
| 20 | Assessment of confounding (eg, comparability of cases and controls in studies where appropriate) | 8-9 |
| 21 | Assessment of study quality, including blinding of quality assessors, stratification or regression on possible predictors of study results | 7-8 |
| 22 | Assessment of heterogeneity | 8-9 |
| 23 | Description of statistical methods (eg, complete description of fixed or random effects models, justification of whether the chosen models account for predictors of study results, dose-response models, or cumulative meta-analysis) in sufficient detail to be replicated | 8-9 |
| 24 | Provision of appropriate tables and graphics | 9 |
| Reporting of results should include | | |
| 25 | Graphic summarizing individual study estimates and overall estimate | Figure 3-4 |
| 26 | Table giving descriptive information for each study included | Table 1 |
| 27 | Results of sensitivity testing (eg, subgroup analysis) | 10-12 |
| 28 | Indication of statistical uncertainty of findings | 10-12 |
| Reporting of discussion should include | | |
| 29 | Quantitative assessment of bias (eg, publication bias) | 12 |
| 30 | Justification for exclusion (eg, exclusion of non-English language citations) | N/A |
| 31 | Assessment of quality of included studies | 14 |
| Reporting of conclusions should include | | |
| 32 | Consideration of alternative explanations for observed results | 15 |
| 33 | Generalization of the conclusions (ie, appropriate for the data presented and within the domain of the literature review) | 15 |
| 34 | Guidelines for future research | 15 |
| 35 | Disclosure of funding source | 1 |

**Appendix B. Literature searching details**

Ovid MEDLINE(R) and Epub Ahead of Print, In-Process, In-Data-Review & Other Non-Indexed Citations, Daily and Versions(R) <1946 to May 25, 2023>

| 1 | exp Troponin T/ or Cardiac Troponin.mp. or exp Troponin I/ | 20358 | 18585 |
| --- | --- | --- | --- |
| 2 | (high-sensitivity or high-sensitive).mp. | 89055 | 77890 |
| 3 | 1 and 2 | 3951 | 3375 |
| 4 | exp Diabetes Mellitus, Type 2/ or exp Diabetes Mellitus/ or diabetes.mp. or exp Diabetes Mellitus, Type 1/ | 786500 | 717258 |
| 5 | 3 and 4 | 255 | 221 |

Embase <1974 to May 25, 2023>

| 1 | exp troponin T/ or exp troponin/ or Cardiac Troponin.mp. or exp troponin I/ | 85912 | 71857 |
| --- | --- | --- | --- |
| 2 | (high-sensitivity or high-sensitive).mp. | 119695 | 103753 |
| 3 | 1 and 2 | 10183 | 8328 |
| 4 | diabetes.mp. or exp diabetes mellitus/ | 1398092 | 1225819 |
| 5 | 3 and 4 | 1306 | 1047 |
| 6 | limit 5 to (embase and (article or article in press or editorial or letter or "review")) | 696 | 531 |
